# Inhibition of miR-22 enhanced the efficacy of icotinib plus pemetrexed in a rat model of non-small cell lung cancer

**DOI:** 10.22038/IJBMS.2019.39291.9320

**Published:** 2020-03

**Authors:** Jing Zhang, Zhi-Qiang Xue, Bin Wang, Jia-Xin Wen, Yun-Xi Wang

**Affiliations:** 1 Department of Thoracic Surgery, The First Medical Center of Chinese PLA General Hospital, Beijing 100853, China

**Keywords:** Carcinoma, Human, Icotinib, MIRN22 microRNA, Non-Small-Cell Lung, Pemetrexed

## Abstract

**Objective(s):**

To investigate the role of miR-22 in the efficacy of combined icotinib (BPI-2009H) and pemetrexed (LY-231514) on tumor growth and apoptosis in rats with non-small cell lung cancer (NSCLC).

**Materials and Methods:**

Rats were injected with HCC827 cells, which were transfected with anti-miR-22, followed by the treatment of BPI-2009H and/or LY-231514. MTT assay was used to detect the inhibition rate of HCC827 cells. qRT-PCR was performed to examine miR-22 expression in HCC827 cells and lung tumor tissues. Moreover, immunohistochemistry and Western blotting were performed to detect the related-molecule expressions, while TUNEL staining was used to observe cell apoptosis of lung tumor tissues.

**Results:**

MiR-22 expression was decreased in HCC827 cells after the treatment of BPI-2009H or LY-231514 in a dose-dependent manner. Both BPI-2009H and LY-231514 increased the inhibition rate of HCC827 cells, which was enhanced by anti-miR-22 with decreased IC50 values. Furthermore, the decreased expression of miR-22 was found after the treatment of BPI-2009H or/and LY-231514 in lung tumor tissues. In addition, the expressions of PCNA, Ki67, and Bcl-2 were reduced, but Bax and Caspase-3 were increased in treated rats, typically in those rats treated with the combination of anti-miR-22, BPI-2009H, and LY-231514.

**Conclusion:**

Inhibition of miR-22 could enhance the efficacy of icotinib combined with pemetrexed in rats with NSCLC, providing a new perspective for NSCLC therapy.

## Introduction

Lung cancer has become one of the most common malignant tumors around the world, among which the number of non-small cell lung cancer (NSCLC) cases accounts for about 80%~85% (1), with over 50% of these patients in the late/advanced stage at the time of diagnosis (2). Chemotherapy has been the main therapy for advanced NSCLC so far, but the prognosis is poor, as recorded, the median overall survival time was shorter than 12 months, and the 5-year survival rate was less than 1% (3). Recent research shows, molecule-targeted medicine with epidermal growth factor receptor (EGFR) as a therapeutic target may effectively improve responses to treatment and prolong progression-free survival (PFS), playing an essential role in the treatment of NSCLC (1, 4, 5), which mainly consists of two types: small-molecule tyrosine kinase inhibitors (TKIs) like afatinib, gefitinib, icotinib, as well as monoclonal antibodies (6). Given the different antitumor effects of mechanism, the combination therapy of EGFR-TKI and chemotherapy has become the hotspot, which could reduce the dose of chemotherapy drugs with decreased overlapping toxicities, and effectively improve the antitumor ability, to be widely used in the clinical treatment of cancers (7, 8).

Pemetrexed is a multi-targeting, anti-metabolite, and anti-folate chemotherapy drug by inhibiting thymidylate synthase (TS), dihydrofolate reductase (DHFR), and several other folate-dependent enzymes, which were implicated in the metabolism and synthesis of DNA precursors, and thereby preventing the formation of DNA and leading to the potent antitumor ability(9, 10). It is of significant advantage to be widely used to treat patients with NSCLC owing to its clinical curative effect as well as low toxic and side effects (11, 12). On the other hand, icotinib, as an effective and specific EGFR-TKI, especially for EGFR-mutant NSCLC, is fat-soluble and safe, and it can easily cross the blood-brain barrier or cell membrane, thus having positive antitumor effects in the clinical treatment of NSCLC (13). Of note, the pemetrexed-based chemotherapy plus icotinib therapy strategy is a promising choice for the treatment of advanced lung cancer (14).

MicroRNAs (miRNAs) are a class of small single-stranded non-coding RNAs with the length of 19-25 bp (15). An increasing number of studies have shown that miRNAs may exert oncogenes or tumor suppressor functions in different types of cancers, which are widely accepted as a diagnostic marker for the evaluation of tumor prognosis and the prediction of treatment efficacy (16, 17). MiR-22, an evolutionarily-conserved gene located in chromosome 17p13, could inhibit lung cancer cell EMT and invasion, thus suppressing lung cancer cell progression (18, 19). It is worthy of mentioning that miR-22 clinically served as a sensitizer in several cancer treatments (20), with the function of increasing chemosensitivity to different anticarcinogens. For example, miR-22 could re-sensitize the paclitaxel-resistant colon cancer cells to paclitaxel (21). Besides, both increased and decreased miR-22 enhanced the fulvestrant sensitivity of the fulvestrant-resistant breast cancer cells (22). Of note, there was a study stating that miR-22 overexpression was correlated with the poor efficacy of pemetrexed therapy in NSCLC patients (23). Furthermore, icotinib was also found capable of suppressing the Akt signaling pathway activation in human NSCLC cells (24), which was associated with miR-22 (25), hypothesizing that miR-22 may affect the icotinib efficacy in NSCLC. However, few studies have investigated whether miR-22 can influence the efficacy of icotinib combined with pemetrexed in the treatment of NSCLC. Therefore, the current research established a rat subcutaneous NSCLC model, injected pLVTHM-anti-miR-22 into the NSCLC rats, with the objective of exploring the inhibition role of miR-22 in the efficacy of BPI-2009H plus LY-231514 on tumor growth and apoptosis in rats with NSCLC.

## Materials and Methods

***Ethics statement***

The animal experiment was approved by our Experimental Animal Ethics Committee of The First Medical Center of Chinese PLA General Hospital, and all procedures were strictly in accordance with relevant provisions of the experimental animal care and use created by the International Association for the Study Pain (26).

***Subjects of study***

Human NSCLC cell lines with an EGFR mutation of HCC827 were purchased from the American Type Culture Collection (ATCC, Manassas, VA, USA). A total of 100 nude male rats were purchased from the Beijing WeitongLihua Experimental Animal Technical Co. Ltd (Beijing, China), and housed in a sterile laminar ﬂow chamber. The rats were fed at normal circadian rhythms, and they could take food and water *ad libitum* in a clean grade animal room at 22~25 t .

***3- (4,5-dimethylthiazol-2-yl)-2,5-diphenyl tetrazolium bromide (MTT) assay***

The effects of Icotinib (BPI-2009H) and Pemetrexed (LY-231514) on the inhibition rate of HCC827 cells were measured using the MTT assay. In brief, Cells (1 × 10^4^) were seeded in 96-well plates followed by adding BPI-2009H (0.01, 0.1, 1, 10, and 100 μmol/l) or LY-231514 (0.01, 0.1, 1, 10, and 100 μg/ml) and MTT solution (5 mg/ml) for incubation at 37 ^°^C for 4 hr. Then, the cells were lysed in 200 μl DMSO, and OD570 was measured with a microplate reader. 

***Establishment of the NSCLC rat models and grouping***

HCC827 cells were digested with 0.25% trypsin and then diluted into a single cell suspension with fresh DMEM medium, which was then centrifuged, washed twice with ice-cold normal saline, and brought to a concentration of 2 × 10^7^ cells/ml. Cell suspension (4 ×10^12^ cells) was inoculated into the subcutis of the back of nude rats using a 1-ml syringe. Animals were then housed in a sterile laminar ﬂow chamber, and changes in tumor growth were observed. After tumors had grown to 1 cm (within 1 week) in diameter, the rats were divided into groups for later experiments. As shown in Table 1, Rats were randomly classified into 10 groups (10 rats per group). Icotinib (BPI-2009H) and Pemetrexed (LY-231514) were purchased from AMQUAR. Lentiviral vector pLVTHM-anti-miR-22 was provided by Hanheng Biotechnology Co., Ltd (Shanghai, China). From the 2nd week, the tumor size of each rat was measured once a week to calculate the volume of tumors (27), and the weight of rats was recorded. After 8 weeks of intervention treatment, rats were anesthetized, and their limbs were fixed to perform normal anatomy. The lung tumor tissue of rats was obtained, half of which was soaked with formalin to make paraffin-embedded tissue blocks, and the other half was preserved in a refrigerator at -80 ^°^C for subsequent experiments.

***qRT-PCR***

The total RNA was extracted according to the instruction of Trizol (Invitrogen, USA), and the purity and concentration were measured by a NanoDrop2000 spectrophotometer (Thermo Scientific, Willmington, DE, USA). cDNA was synthesized using a stem-loop specific primer for miR-22 and then subjected to qRT-PCR using 2 μl of a 1:5 dilution of the reverse-transcribed cDNA and SYBR green in an ABI Fast Q-PCR machine (Applied Biosystems, Foster City, CA, USA). The cycling conditions were as follows: 50 ^°^C for 2 min, 95^ °^C for 5 min, and 40 cycles of 95 ^°^C for 15 sec followed by 60 ^°^C for 1 min. Based on the gene sequences published in the Genbank database, the primers were designed with the software Primer 5.0 and synthesized by Shanghai Sangon Biotechnology Co. Ltd (Table 2). U6 was used as the internal reference gene and 2^-Δ Δ Ct ^was applied to present the relative expression of miR-22. The experiment was repeated three times.

***Immunohistochemistry***

Lung tissues were embedded in paraffin and sliced into 4 μm sections, which were baked and immersed into a newly-prepared xylene solution for 10 min twice. Then, dewaxing was performed with gradient alcohol for 5 min. After washing with water, slides were incubated for 10 min with 3% H_2_O_2_ at 37 ^°^C. Next, sections were blocked for 30 min with normal goat serum, washed for 5 min × 3 times with PBS, and primary antibody solution (PCNA: 1:10000 dilution, ab29; Ki67: 1:100 dilution, ab16667, all purchased from Abcam, Cambridge, MA, USA) added for overnight reaction at 4 ^°^C. Then, a secondary antibody was added for 30 min at 37 ^°^C, and cells were washed with PBS three times within 30 min at 37 ^°^C. Later, color development was performed with DAB for 20 min under a light microscope and terminated with PBS before routine dehydration with ethanol, hyalinization with xylene, and section sealing with neutral resin. Two independent investigators examined all tumor sections randomly. Five views were examined per section, and 100 cells were observed per view at 400 ×magnification. The number of positive cells was counted, and the proliferation index (PI) of PCNA and Ki67 was calculated, with the formula PI = number of positive cells/total cell number× 100% (28). The experiment was repeated three times.

***Western blotting***

Total protein in lung tissues was extracted, and its concentration was determined according to the instructions of the BCA Kit (Wuhan BOSTER Biological Technology Co., Ltd, China). Then, loading buffer was added into the extracted protein samples to boil for 10 min at 95 ^°^C before loading samples 40 ug/hole. Next, 10% polyacrylamide gel was used to separate proteins by electrophoresis with the voltage of 80V for concentration gel and 120V for separation gel. The wet transfer was utilized with constant 100 mV for 90–120 min of PVDF membrane transferring. After blocking with 5% BSA at room temperature for 1 hr, primary antibodies PCNA (1 µg/ml, ab29), Ki-67(1 µg/ml, ab16667), Bax (1:1000 dilution, ab32503), Bcl-2 (1:1000 dilution, ab32124), Caspases-3 (1:500 dilution, ab2171), and β-actin(1:10000 dilution, ab8226) (all purchased from Abcam, Cambridge, MA, USA) were added for overnight incubation at 4 °C. Next, cells were washed with TBST for 5 min × 3 times before adding corresponding secondary antibodies for another 1 hr of incubation. Last, cells were washed again with TBST for 5 min × 3 times before developing with chemiluminescence reagent. β-actin was used as the loading control, and the experiment was repeated three times.

***TUNEL staining***

Lung tissue samples were prepared into frozen sections, which were dried at room temperature and blocked for 1 hr using 3%BSA Tris-Hcl (Roche). Then, sections were washed with PBS and 50 μl of TUNEL reaction mixture were added for 1 hr of incubation at 37 °C, 3% H_2_O_2 _to block endogenous peroxidase, as well as peroxidase-labeled fluorescence antibody for 30 min of incubation. Next, after washing three times with PBS, sections were mounted with glycerol and placed under an OLYMPUS fluorescence microscope for observation and picture taking. The number of TUNEL staining positive cells was counted and the rate of apoptotic cells was calculated with the formula: rate of apoptotic cells = number of apoptotic cells/ total cell number × 100%. The experiment was repeated three times.

***Statistical method***

The statistical data were analyzed using SPSS 22.0 and GraphPad Prism 6.0 software packages. Measurement data were presented by mean ± standard deviation (±s). One-way ANOVA was used for differences between multiple groups followed by Tukey’s HSD (honestly significant difference) test to compare the difference between two groups. The IC_50_ values were calculated using GraphPad Prism 6.0 with the nonlinear regression curve fit. Survival analysis was performed using the Kaplan-Meier curve*. P*<0.05 was considered statistically different.

## Results

***Both BPI-2009H and LY-231514 increased the inhibition rate of HCC827 cells, which was enhanced by anti-miR-22***

**Figure 1:**
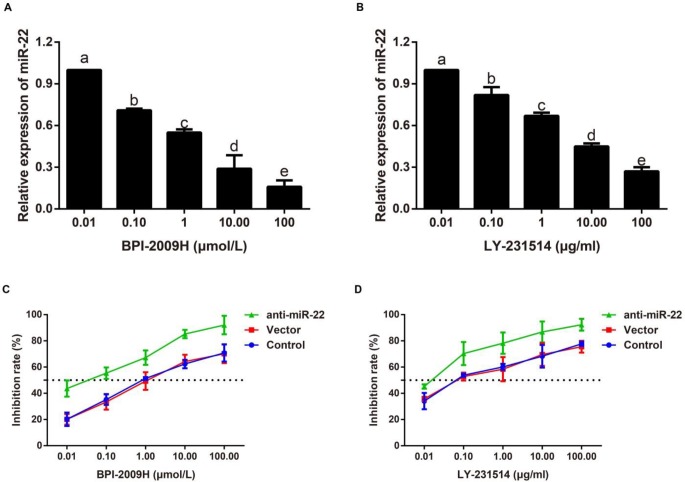
BPI-2009H and LY-231514 increased the inhibition rate of HCC827 cells, which was enhanced by anti-miR-22

Note: qRT-PCR was used to detect the expression of miR-22 in HCC827 cells treated with BPI-2009H (A) and LY-231514 (B), Same letters showed no significant difference, *P*>0.05; different letters showed the statistically significant difference, *P*<0.05; C-D: anti-miR-22 increased the inhibition rate of HCC827 treated with BPI-2009H (C) and LY-231514 (D)

As demonstrated in Figures 1A-B, miR-22 expression was significantly decreased in HCC827 cells after treatment of BPI-2009H or LY-231514 in a dose-dependent manner. Besides, the IC_50_ values of BPI-2009H and LY-231514 on HCC827 cells were 1.67±0.33 μmol/l and 0.74±0.09 μg/ml, respectively, which were decreased by the transfection of anti-miR-22 with IC_50_ values of 0.064±0.004 μmol/l and 0.014±0.002 μg/ml, respectively (Figures 1C-D). 

***General information of rats***

**Figure 2:**
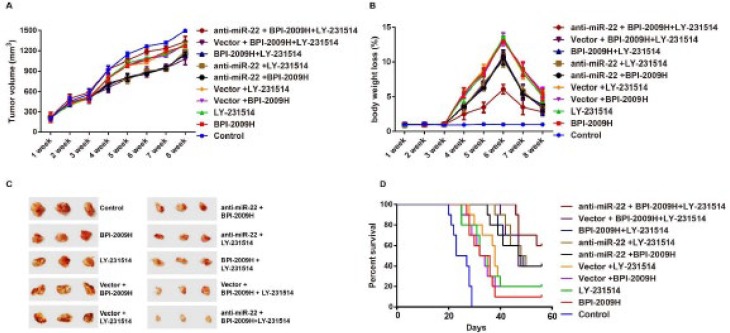
General information of rats

Note: A-B, The tumor volume (mm3) and bodyweight loss (%) of rats in each group from one week after subcutaneous tumor inoculation; C: Image of representative tumors collected from rats at the end of the experiment; D: Survival curves of rats in each group

One week after subcutaneous tumor inoculation, all rats had solid tumors (100% tumor formation rate), and the tumor volume showed no significant difference between groups (all *P*>0.05). From the beginning of the 2^nd^ week, compared with the Control group, the tumor growth of rats in the other groups was inhibited to some extent. And the obvious differences of tumor volume and bodyweight loss were observed since the 4^th^ week between groups (all *P*<0.05). Changes in the tumor volume and bodyweight loss are demonstrated in Figures 2A-C. Besides, significant difference in the prognosis of rats was found between the groups with the highest survival rate in anti-miR-22 + BPI-2009H + LY-231514 group (Figure 2D, *P*<0.001).

***Expression of miR-22 in lung tumor tissues of rats in each group***

**Figure 3:**
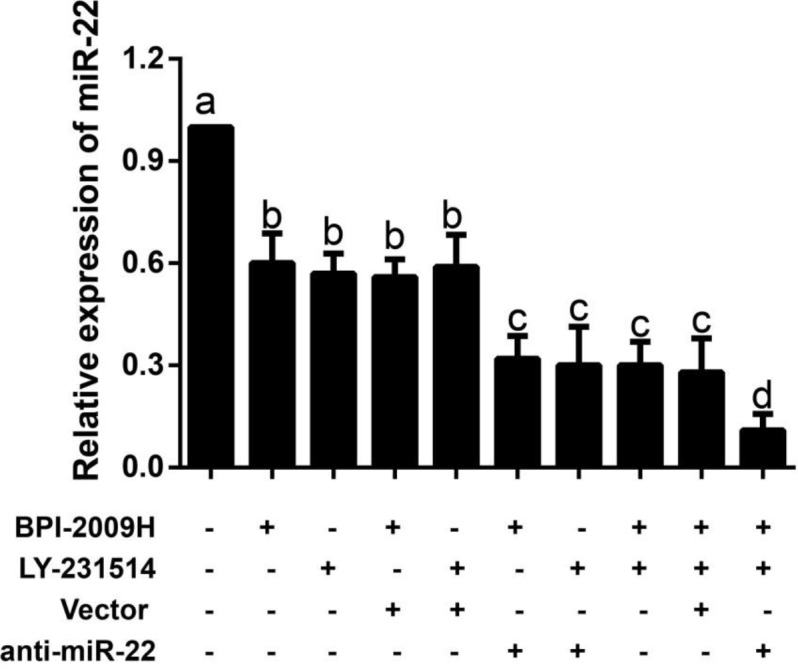
qRT-PCR was used to detect the expression of miR-22 in lung tumor tissues of rat

Note: Same letters showed no significant difference, *P*>0.05; different letters showed statistically significant difference,* P*<0.05

According to the qRT-PCR results, anti-miR-22 + BPI-2009H + LY-231514 group showed the lowest expression of miR-22 in lung tumor tissues among those ten groups (all *P*<0.05). In addition, as compared with the Control group, BPI-2009H and LY-231514 groups had a remarkably lowered expression of miR-22. Moreover, the combination treatment of BPI-2009H and LY-231514 decreased the miR-22 expression when compared with the treatment of BPI-2009H or LY-231514 alone (both *P*<0.05, Figure 3).

***Expression of PCNA and Ki67 in lung tumor tissues of rats in each group***

**Figure 4.**
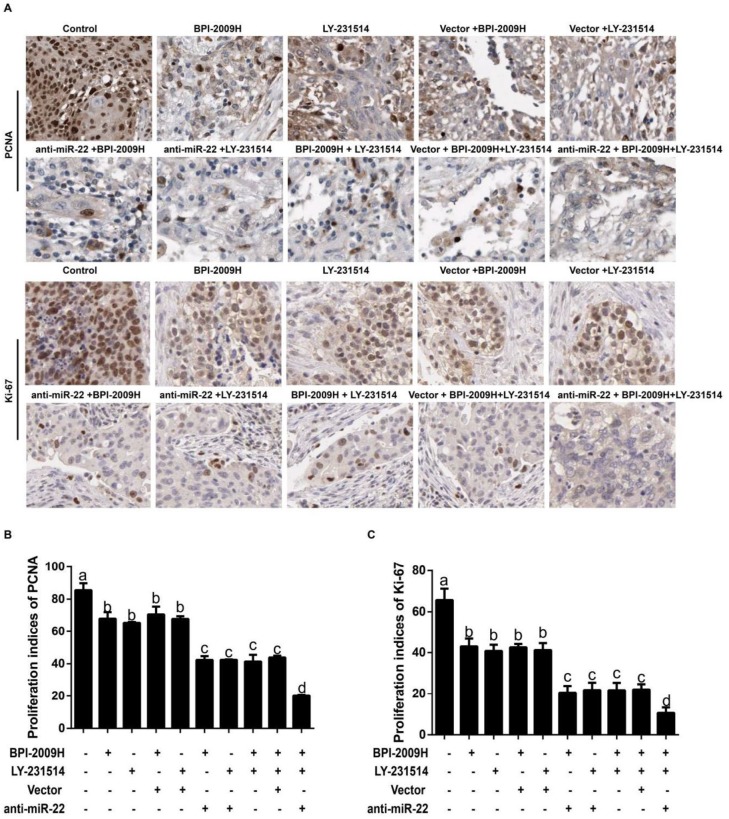
The expression of PCNA and Ki67 in lung tumor tissues of rats in each group detected by immunohistochemistry staining

Note: A, Representative sections stained for PCNA and Ki67 detected by immunohistochemistry staining; B–C, The statistical analysis of the proliferative indices (PIs) of PCNA (B) and Ki-67 (C) in lung tissues; same letters showed no significant difference, *P*>0.05; different letters showed statistically significant difference,* P*<0.05

**Figure 5:**
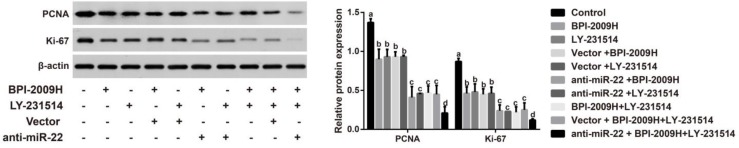
Protein expressions of PCNA and Ki-67 in lung tumor tissues of rats in each group by Western blotting

Note: Same letters showed no significant difference, *P*>0.05; different letters showed statistically significant difference, *P*<0.05

Immunohistochemical staining showed positive PCNA and Ki67 expression (brown-yellow particles) localized in the nuclei (Figure 4A). By comparison with the Control group, the proliferative indices (PIs) of PCNA and Ki67 in lung tumor tissues of other groups were significantly decreased (all *P*<0.05). Further, when compared with BPI-2009H + LY-231514, the PIs of PCNA and Ki67 were obviously lowered in the anti-miR-22+BPI-2009H + LY-231514 group (both *P*<0.05, Figures 4B-C). Besides, Western blotting was also performed to measure the protein expression of PCNA and Ki-67 in lung tumor tissues (Figure 5), which presented to be consistent with those of immunohistochemistry staining. 

***Cell apoptosis in lung tumor tissues of rats in each group***

**Figure 6.**
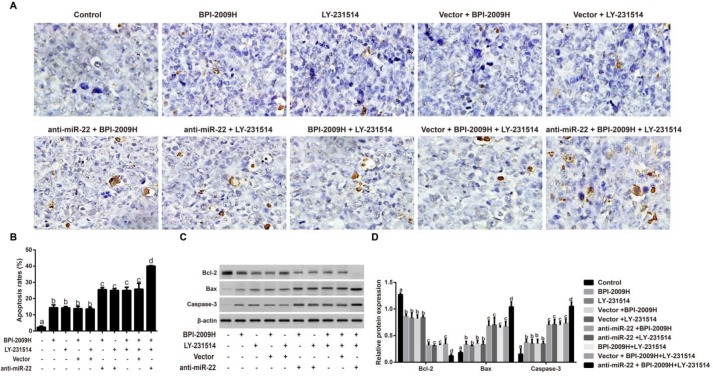
Cell apoptosis in lung tumor tissues of rats in each group

Note: A, Representative sections stained for apoptosis by TUNEL assay; B, The cell apoptosis rate in lung tumor tissues in each group; C-D, The protein expressions of Bax, Caspase-3, and Bcl-2 in lung tumor tissues of rats in each group detected by Western blotting. Same letters showed no significant difference, *P*>0.05; Different letters showed statistically significant difference, P<0.05. TUNEL, terminal deoxynucleotidyl transferase-mediated uridine 5’-triphosphate-biotin nick end labeling

When compared with the Control group, the cell apoptosis rate in lung tumor tissues of rats in the rest of the groups was significantly increased with the up-regulation of Bax and Caspase-3 expression, as well as the down-regulation of Bcl-2 expression (all* P*<0.05). Moreover, rats in the anti-miR-22 + BPI-2009H + LY-231514 group had a notably higher apoptosis rate, obviously increased expression of Bax and caspases-3, as well as decreased expression of Bcl-2 (all *P *<0.05, Figure 6).

## Discussion

To date, the combination of chemotherapy and EGFR-TKIs has become a better choice in NSCLC, and we have carried out this study to determine the role of miR-22 in the efficacy of combined therapy in NSCLC rat model. Our results suggest that expression of miR-22 was decreased in HCC827 cells and NSCLC rats after treatment with BPI-2009H and/or LY-231514, and the tumor growth was significantly inhibited with prolonged survival rate and less bodyweight loss, especially evident in NSCLC rats with intratumoral injection of anti-miR-22, which could further improve the efficacy to a large extent. As is known to all, pemetrexed is a kind of multi-target anti-folic agent that can directly inhibit the activity of key enzymes during the metabolism of folic acid, thus inhibiting the growth of tumors (29). Meanwhile, miRNAs have been demonstrated to affect responses to pemetrexed through targeting key enzymes related to folate pathway proteins (30). For example, dihydrofolate reductase, as the target enzyme of inhibition by pemetrexed, was found to be modulated by miR-24 at the translational level (31). To our knowledge, miR-22 could directly target methylenetetrahydrofolate reductase (MTHFR), one of the critical enzymes in the metabolism of folic acid (32, 33). The gene polymorphism of *MTHFR* was found to be associated with the efficacy of pemetrexed therapy in patients with NSCLC (34), and 677C > T SNP in the *MTHFR* gene can lead to reduced expression of MTHFR as well as lowered levels of 5-methylTHF, resulting in the enhanced activity of TS and the decreased therapeutic effect of pemetrexed (35). These may provide an explanation that miR-22 may affect the curative efficacy of pemetrexed in patients with NSCLC by interfering with the metabolism of folic acid. Consistently, Franchina *et al*. also revealed that the expression of miR-22 was significantly reduced in NSCLC patients after treatment with pemetrexed (23). On the other hand, icotinib was also found capable of suppressing the Akt signaling pathway activation in human NSCLC cells (24), and over-expressed miR-22 can down-regulate phosphatase and tensin homolog and activate phosphoinositide 3-kinase (PI3K)/AKT pathway (25), showing that miR-22 may affect the icotinib efficacy in NSCLC through the Akt signaling pathway. Given the facts above, we hypothesized that inhibition of miR-22 might affect the therapeutic effect of icotinib plus pemetrexed through different mechanisms, thus enhancing their combined efficacy in the treatment of NSCLC.

Now that the main molecular mechanism of malignant tumors is the unrestricted proliferation and apoptosis reduction of tumor cells, inhibiting the malignant proliferation and inducing the apoptosis of tumor cells has become a hot topic for finding a new treatment for tumors (36, 37). Ki67 and PCNA, the most widely used proliferation labeling proteins, are expressed in the nucleus and closely related to cell cycles (38), which are more frequently down-regulated in good responders to treatment. As suggested by Horii *et al.* obvious decreases in Ki-67 and PCNA labeling indexes (LIs) were discovered in the surgical specimens of patients with esophageal squamous cell carcinoma who had neoadjuvant chemotherapy compared with those patients without preoperative treatment (39). Therefore, the expressions of Ki67 and PCNA were detected by immunohistochemistry, generally used clinically, in this study (40), and our result confirm that after the combined icotinib and pemetrexed treatment, the PIs of PCNA and Ki67 in the NSCLC rats injected with anti-miR-22 were appreciably lower than other resting groups, which is also consistent with our findings by using Western blotting. More importantly, some miRNAs can induce tumor cell proliferation, thereby reducing the sensitivity of drugs, such as miR-96, which can promote cell proliferation by targeting RECK and lower the chemotherapy sensitivity of esophageal cancer cells(41). Therefore, we hypothesized that inhibition of miR-22 might inhibit the proliferation of NSCLC cells and enhance the therapeutic effects of icotinib plus pemetrexed, thus affecting tumor growth. At the same time, mounting studies have proved the great significance of cell apoptosis dysfunction in the resistance to anticancer drugs, including icotinib and pemetrexed. Icotinib could lead to Tca8113 cell apoptosis possibly due to interfering with the reactive oxygen species-mediated MAPK pathway (42). Besides, by activating ataxia telangiectasia mutated (ATM)/p53-dependent and -independent signaling pathways, pemetrexed can promote both intrinsic as well as extrinsic apoptosis (43). Moreover, miRNAs may also be involved in the promotion of cell apoptosis combined with drugs. Tang *et al*. and his team reported that knockdown of the miR-183/96/182 cluster could improve the effect of Temozolomide in treating glioma by affecting the ROS-mediated apoptosis pathway (44). Similar to our findings, apoptosis-inhibited molecules (Bcl-2) were apparently lowered, but pro-apoptotic factors including Bax and Caspase-3 were greatly elevated in treated NSCLC rats with anti-miR-22, indicating that inhibition of miR-22 may promote apoptosis to increase the therapeutic outcomes and suppress the tumor growth. 

**Table 1:** Intervention treatments of all groups after the successful establishment of subcutaneous tumor inoculation

Group	The 2^nd^ week	The 3^rd^ week
Control	Injection (IT) of normal saline	Oral and injection (IP) of normal saline, 5 weeks
BPI-2009H	Injection (IT) of normal saline	BPI-2009H (oral 60 mg/kg/day), 5 weeks
LY-231514	Injection (IT) of normal saline	Injection (IP) of LY-231514 (100 mg/kg/day), 5 weeks
Vector +BPI-2009H	Injection (IT) of Vector (1nM)	BPI-2009H (oral 60 mg/kg/day), 5 weeks
Vector +LY-231514	Injection (IT) of Vector (1nM)	Injection (IP) of LY-231514 (100 mg/kg/day), 5 weeks
anti-miR-22 +BPI-2009H	Injection (IT) of pLVTHM-anti-miR-22 (1nM)	BPI-2009H (oral 60 mg/kg/day), 5 weeks
anti-miR-22 +LY-231514	Injection (IT) of pLVTHM-anti-miR-22 (1nM)	Injection (IP) of LY-231514 (100 mg/kg/day), 5 weeks
BPI-2009H + LY-231514	Injection (IT) of normal saline	BPI-2009H (oral 60 mg/kg/day) and injection (IP) of LY-231514 (100 mg/kg/day), 5 weeks
Vector + BPI-2009H + LY-231514	Injection (IT) of Vector (1nM)	BPI-2009H (oral 60 mg/kg/day) and injection (IP) of LY-231514 (100 mg/kg/day), 5 weeks
anti-miR-22 + BPI-2009H + LY-231514	Injection (IT) of pLVTHM-anti-miR-22 (1nM)	BPI-2009H (oral 60 mg/kg/day) and injection (IP) of LY-231514 (100 mg/kg/day), 5 weeks

IT: intratumoral; IP: intraperitoneal

**Table 2:** Primers sequences of quantitative reverse transcription-PCR (qRT-PCR)

Gene		Sequence
miR-22	Forward	5’-ACACTCCAGCTGGGAAGCTGCCAGTTGAAG-3’
	Reverse	5’-GGTGTCGTGGAGTCGGCAA-3’
U6	Forward	5’-CTCGCTTCGGCAGCACATATACT-3’
	Reverse	5’-ACGCTTCACGAATTTGCGTGTC-3’

## Conclusion

Inhibition of miR-22 may inhibit cell proliferation and promote cell apoptosis to enhance the efficacy of combined icotinib & pemetrexed, thereby inhibiting the tumor growth of NSCLC. However, the mechanism of miR-22 in modulating the gene network or downstream pathways to affect the efficacy of combined therapy needs to be further explored in future studies.
